# A Thesis on the Action of Remedies

**Published:** 1880-04

**Authors:** C. C. Maddox


ATLANTA
Medical and Surgical Journal.

VOL. XVIII.] APRIL—1880. [No. 1.

### Original Communications.

### A THESIS, ON THE ACTION OF REMEDIES, PRESENTED TO THE FACULTY OF ATLANTA MEDICAL COLLEGE, MARCH, 1880.

BY C. C. MADDOX.

To whom was awarded a medical case, the prize offered for the best Thesis on this subject.

There is, perhaps, no subject within the range of medical science that is so essential to the successful treatment of disease, and with which the student and practitioner should be more thoroughly acquainted, than the one which we are now about to consider. In order to treat diseases rationally and successfully, we must possess an accurate knowledge of the cause and nature of the pathological conditions upon which our remedies are to act, as well as the physiological action of those remedies. It is important that we be able to distinguish the difference between the physiological action of a remedy and its therapeutical result. They bear the same relation to each other as cause and effect.

The physiological action of a remedy is that action which is exerted upon an organ or tissue whether it be in a diseased or healthy condition. And this being the only true action of a remedy, a thorough understanding of it is strictly necessary to the proper treatment of disease, and to discard the erroneous idea of specifics for the cure of diseases. According to the physiological classification of remedies we have two grand divisions, namely—local and elective. Local remedies act directly upon the part to which they are applied. For instance, substances placed upon the integument with the view of producing counter-irritation, or placed in contact with inflamed and irritated surfaces to soothe and defend them act locally. Certain agents are also introduced into the stomach for their local action upon its surface. For example, demulcents and astringents are thus administered when the mucous membrane of this organ requires their impression. In some instances elective and local remedies may be applied to the same part, but for very different purposes. In one, absorption is necessary before its effect is realized ; the other is intended to affect the surface to which it is applied. Some agents have both local and elective properties, affecting the part to which they are applied, and entering the circulation, affect other parts also. Thus, tartarized antimony when applied to the skin, exerts its local action as a pustulant, while, if it be absorbed, will act electively as a powerful emetic and cardiac sedative. Elective remedies make their impression upon the organ of their selection, only by being carried by the blood or other fluid to that organ or tissue. The term elective given to this grand division of remedies is used instead of general or constitutional for the reason it implies their preference for certain organs, and because these names convey the idea that some agents act directly upon every part of the body, when there is, perhaps, no one remedy that has such influence. Some elective remedies by exerting a powerful influence upon an important or vital organ may in this indirect manner affect several others at the same time. For instance, any remedy administered which would modify the action of the brain in any way very much, it presiding over the whole organism, would, in this way, affect the function of some other organs to a greater or less extent, but that change would only be the result of the remedy's action upon the brain.

All remedies act in one of three ways : either mechanically, chemically or vitally. It is through mechanical process that demulcents protect denuded and inflamed surfaces externally and internally, from the air or other irritating causes. It can be by no other than mechanical process that wheat bran and coarsely pulverized charcoal correct the sluggish and inactive peristaltic muscular contractions of the stomach and bowels. Agents are said to act through chemical process when their action cannot be explained upon any other hypothesis than a chemical affinity existing between the remedy and the fluids and tissues with which it comes in contact, and upon which its power is exerted. The action of hæmostatics upon the blood causing its ready coagulation, so as to arrest hemorrhage, is the result of chemical change wrought in that fluid. Escharotics, antiseptics and various other agents produce their effect through chemical process. By vital process is meant those changes which are produced in the capillary circulation, or modification of the vitality of a part without any mechanical irritation or chemical reaction taking place with the solids or fluids of the part.

Some remedies, both local and elective, have two actions, known as primary and secondary actions. The primary action of an agent is the first change or impression made upon an organ or tissue by it. Opium acts upon the brain, the great controlling nervous centre—first as a powerful stimulant which is soon followed by the secondary and more lasting impression which is sedative. Most stimulants leave the function of the organs upon which they act in a more or less depressed condition after the first or exciting effect has passed off. The secondary action follows, and is very probably the result of the first effect of the remedy.

The therapeutical results of these physiological actions may be direct or indirect. A direct therapeutic result is found where the curative effect is produced in the part upon which the remedy acts.

The result is indirect when the curative change is had in an organ or tissue other than the one upon which the physiological action of the remedy is exerted.

There are various influences and conditions which modify very considerably the ordinary action of remedies, and it is quite important that the medical practitioner be familiar with and remember these circumstances in treating diseases, for, without such knowledge, he may experience unexpected results.

1. Climate is generally thought to modify, to a considerable extent, the physiological action of certain remedies; thus, people of the North are said to be less susceptible to the action of opium than those who live in a more heated situation. The reverse is true as regards mercurials, especially when we look to their elective and exciting influence upon the liver and salivary glands. Calorifics are required in less quantity to sustain the natural and normal temperature than in colder latitudes.

2. Habit—This has a decidedly modifying influence upon the action of certain remedies, and should not be lost sight of in their administration. For instance, take an old toper, whose brain has become accustomed to stimulation, from the use of alcoholic liquors, and it will require a much larger quantity to produce this effect, than for a person who is more temperate. To the opium eater we can without hesitation and without any unpleasant symptoms, administer morphia in doses that would prove injurious and perhaps destructive to persons unaccustomed to its use. In most instances, habitual use renders the system less susceptible to the action of some remedies, while with others, the contrary result is observed.

3. Age, perhaps, exercises a more decided influence upon the effects of remedies than any other circumstance, independently of the proportionate quantities for different periods of life as compared to the adult dose. For while a child requires less opium in proportion to the grown person, mercurials may be administered in much larger quantities without any unpleasant consequences. An amount of mercury that would likely be sufficient to produce ptyalism in the adult, will affect no such results upon the child.

4. Sex—In the majority of instances, we may administer medicines to both sexes in the same doses, but, owing to their greater sensitiveness to nervous impressions, narcotics should be given to females cautiously. Another and perhaps more important consideration under this head, is that of the uterine function. The changes that the system undergoes in consequence of that physiological condition—pregnancy—to which the woman is subject, modify greatly the action of remedies, and very frequently prevent the physician from adopting the course of treatment that he would otherwise pursue to correct certain morbid conditions.

5. Idiosyncrasy—This individual peculiarity evidently modifies in a great degree, the ordinary physiological action of medicines and should always be ascertained and remembered in prescribing.

6. Incompatibility—This undoubtedly changes greatly the ordinary action of certain remedies; for some of our most active and valuable agents when combined, become worthless and consequently useless in the treatment of disease, while on the other hand, certain remedies that are perfectly harmless by themselves, in certain combinations are virulent poisons.

There are many conditions which influence more or less the natural action of remedies, but we have deemed it only necessary to mention in this connection those of greatest importance.

When the student of medicine has completely mastered, and has practically familiarized himself with the direct physiological action of remedies, even to the most minute detail, he has satisfied the demands of humanity—has done all within the reach of man to mitigate the suffering, alleviate the pains and relieve the diseases to which humanity is heir.

To aid the medical student in this great achievement, Dr. J. G: Westmoreland has performed a labor reflecting much credit upon himself and conferring a lasting favor upon the profession. His single volume is a masterpiece of lucid condensation and a general grasp of an enormously wide subject. Its precision of language, clearness of method, completeness of analysis and logical fertility, place it within easy grasp of any one. We will venture to believe that it is essentially perfect in its adaptations.

It is a superb monument to the genius of its faithful architect. Especially does it signalize the cultivated taste and rare practical skill of that one who supervised its construction. Long may it remain a blessing to successive generations; and long may the spirit of fitness and order and majestic simplicity, here so visibly enwrought, be felt as a salutary influence, chastening and elevating in the practice and conflicts of the profession.

